# The Complex Interplay Between Trait Fatigue and Cognition in Multiple Sclerosis

**DOI:** 10.5334/pb.1125

**Published:** 2022-03-16

**Authors:** C. Guillemin, E. Lommers, G. Delrue, E. Gester, P. Maquet, F. Collette

**Affiliations:** 1 GIGA-CRC In Vivo Imaging, University of Liège, Liège, Belgium; 2 Psychology and Cognitive Neuroscience Research Unit, University of Liège, Liège, Belgium; 3 Department of Neurology, CHU of Liège Sart Tilman, Liège, Belgium

**Keywords:** Multiple Sclerosis, Cognition, Fatigue, Anxiety, Depression

## Abstract

Cognitive impairments are frequent in patients with Multiple Sclerosis (MS). Yet, the influence of MS-related symptoms on cognitive status is not clear. Studies investigating the impact of trait fatigue along with anxio-depressive symptoms on cognition are seldom, and even less considered fatigue as multidimensional. Moreover, these studies provided conflicting results.

Twenty-nine MS patients and 28 healthy controls, matched on age, gender and education underwent a full comprehensive neuropsychological assessment. Anxio-depressive and fatigue symptoms were assessed using the HAD scale and the MFIS, respectively. Six composite scores were derived from the neuropsychological assessment, reflecting the cognitive domains of working memory, verbal and visual learning, executive functions, attention and processing speed. Stepwise regression analyses were conducted in each group to investigate if trait cognitive and physical fatigue, depression and anxiety are relevant predictors of performance in each cognitive domain. In order to control for disease progression, patient’s EDSS score was also entered as predictor variable.

In the MS group, trait physical fatigue was the only significant predictor of working memory score. Cognitive fatigue was a predictor for executive functioning performance and for processing speed (as well as EDSS score for processing speed). In the healthy controls group, only an association between executive functioning and depression was observed.

Fatigue predicted cognition in MS patients only, beyond anxio-depressive symptoms and disease progression. Considering fatigue as a multidimensional symptom is paramount to better understand its association with cognition, as physical and cognitive fatigue are predictors of different cognitive processes.

## Introduction

Multiple Sclerosis (MS) is a chronic inflammatory disease resulting from immune-mediated damage in the central nervous system. It is characterized by demyelination and axonal damage to white and grey matters, and leads to various neurologic symptoms (Calabrese et al., 2015; Kutzelnigg et al., 2005). Three different clinical courses are usually recognized (Lublin & Reingold, 1996; Reich, Lucchinetti, & Calabresi, 2018). The relapsing-remitting form of MS (RRMS) is characterized by acute neurologic relapses, followed by total or partial remission. Eventually, the disease can evolve into a progressive form, known as secondary progressive multiple sclerosis (SPMS), during which a progressive and constant increase of disease burden occurs with or without any clearly clinically identified relapses. A minority of patients present a progressive course from disease onset, namely a primary progressive disease course (PPMS).

Motor and sensory deficits are among the most common symptoms of the disease. However, cognitive impairments are also frequent, since they are experienced in about half of the patients (Benedict & Bobholz, 2007) and are sometimes observed after the first demyelinating episode (DiGiuseppe, Blair & Morrow, 2018). The spatial dissemination of grey and white matter lesions results in a large heterogeneity in cognitive deficits across patients (Chiaravalloti & DeLuca, 2008). Yet, reduced processing speed is the most frequent impairment, observed in one third of patients as early as after the first year following a diagnosis of RRMS (DiGiuseppe et al., 2018). Beyond information processing speed, memory (learning and retrieval of new information, in both verbal and visual modalities) is commonly affected in MS, whereas deficits in executive functioning, working memory, attention and visual perception, albeit frequently observed, are less common (see Benedict, Amato, DeLuca & Geurts, 2020 and Chiaravalloti & DeLuca, 2008 for reviews).

The determinants of MS-related neuropsychological impairments are only partially identified. While the severity of cognitive impairments is not linked to disease duration (Beatty, Goodkin & Monson, 1990; Lynch, Parmenter & Denney, 2005), it is determined by the course of the disease. A recent meta-analysis observed that PPMS patients are more severely cognitively impaired than RRMS patients, independently of demographic characteristics (sex, education, disease duration), manual dexterity and physical disability (Johnen et al., 2017). At the brain level, lesion load, grey matter atrophy and third ventricle volume most consistently predict the cognitive status (Arnett et al., 1994; Benedict et al., 2006; Brass, Benedict, Weinstock-Guttman, Munschauer & Bakshi, 2006; Houtchens et al., 2007; Patti et al., 2009; Pinter et al., 2014; Rocca et al., 2018; Sanfilipo, Benedict, Weinstock-Guttman & Bakshi, 2006; Tekok-Kilic et al., 2007). Yet, brain abnormalities alone do not fully explain cognitive impairments in MS (Amato, Zipoli & Portaccio, 2006; Hoffmann, Tittgemeyer & von Cramon, 2007).

Depressive symptoms and anxiety also influence cognition in MS. For instance, patients with anxio-depressive symptoms are four times more likely to show cognitive deficits (Kalron, Aloni & Allali, 2018). Depressive symptoms negatively influence processing speed, executive functioning, attention or memory (DiGiuseppe et al., 2018; Golan et al., 2018; Morrow, Rosehart & Pantazopoulos, 2016; Nunnari et al., 2015) while anxiety alters processing speed, working memory, visuo-spatial memory and/or verbal learning abilities (Morrow et al., 2016; Vissicchio et al., 2019; Whitehouse et al., 2019). However, depression and anxiety only partially explain cognitive impairment in MS and the cognitive domains to which they are associated can largely differ across studies.

Finally, fatigue is also a major symptom of MS that might have an impact on cognition. Seventy-five to 86% of MS patients experience debilitating fatigue that interferes with daily living activities (Bakshi et al., 2000; Krupp, Alvarez, LaRocca, Scheinberg & And, 1988.; Lerdal, Gulowsen Celius, Krupp & Dahl, 2007; Rooney, Wood, Moffat & Paul, 2019; Weiland et al., 2015). When fatigue is acute, follows an intense and/or prolonged effort, it is usually dubbed ‘state fatigue’. By contrast, ‘trait fatigue’ refers to a feeling that persists along the day, does not necessarily follow effort and persists after rest. Fatigue can occur at any stage of the disease, regardless of disease course (Weiland et al., 2015), although patients presenting a progressive phenotype are more at risk and experience a greater severity of fatigue (Rooney et al., 2019). However, fatigue cannot be attributable to the sole chronicity of the disease, as MS patients reported a more frequent and severe fatigue than hypertensive patients (Fisk, Pontefract, Ritvo, Archibald, & Murray, 1994). Despite its incidence, the origin of MS-related fatigue remains unclear (see Kos, Kerckhofs, Nagels, D’hooghe, & Ilsbroukx, 2008 and Penner & Paul, 2017 for reviews). While the influence of factors such as sleep disorder, depression or medication is well-establish, results are still conflicting regarding its pathogenesis. Focal and diffuse brain alterations, pro-inflammatory cytokines release, disturbed activity of the HPA axis, dopamine imbalance and functional cerebral reorganization are among the most studied contributing factors in relation to primary fatigue. Finally, MS-related fatigue differs from fatigue experienced by healthy subjects in daily life. Indeed, patients consider that their fatigue has changed since the disease outbreak (Krupp et al., 1988). When MS-patients are asked about fatigue symptom, they differ from healthy controls by describing fatigue as arising more easily, causing frequent problems and interfering with their responsibilities and physical functioning (Krupp et al., 1988). Consequently, fatigue is one of the principal cause of impaired quality of life in MS (Braley & Chervin, 2010), and more than half of the patients consider fatigue as one of the worst symptoms of the disease (Fisk, Pontefract, Ritvo, Archibald & Murray, 1994).

Most studies interested in the link between fatigue and cognition in MS used a fatigue induction protocol and tried to evidence an association between the rise of subjective fatigue and the decrement of performance. These protocols allow to specifically explore the link between state fatigue and fatigability (see for example Borragán et al., 2018 and Hu, Muhlert, Robertson, & Winter, 2019). The relationship between trait fatigue and cognitive deficit, however, has been less studied and is still debated. Yet this is paramount, as trait fatigue could have an impact on daily living besides fatiguing and effortful situations. Studies investigating the link between fatigue and cognition found an association (Andreasen et al., 2019; Diamond, Johnson, Kaufman & Graves, 2008; Heesen et al., 2010; Pokryszko-Dragan et al., 2016), but not systematically (Bailey, Channon, & Beaumont, 2007; Hildebrandt & Eling, 2014; Krupp & Elkins, 2000; Morrow, Weinstock-Guttman, Munschauer, Hojnacki, & Benedict, 2009; Niino et al., 2014; Parmenter, Denney, & Lynch, 2003). When the influence of confounding factors was taken into account (such as depression, apathy, pharmacological treatment and/or level of disability), the association remained significant in some studies (Andreasen et al., 2019; Diamond et al., 2008; Heesen et al., 2010; Pokryszko-Dragan et al., 2016) but not in others (Golan et al., 2018; Jougleux-Vie et al., 2014; Niino et al., 2014).

Surprisingly, few of these studies distinguished and assessed simultaneously the effect of cognitive and physical fatigue on neuropsychological deficits. These studies reported that both cognitive and physical fatigue are associated to impaired attention and processing speed, yet to a lesser extent for physical fatigue (Andreasen et al., 2019; Heesen et al., 2010; Pokryszko-Dragan et al., 2016). When controlling for confounding factors such as depression and disability, these relationships become, however, less clear (Andreasen et al., 2019; Jougleux-Vie et al., 2014). In the absence of matched healthy controls (HC), the specificity of the relationship between subjective feeling of fatigue and cognition in MS remains uncertain.

To sum up, there is no clear evidence at this time on how fatigue influences cognition in MS, and to which extent this association is explained by the anxio-depressive status. Moreover, few studies tried to disentangle the effects of cognitive and physical fatigue. A better understanding of these clinical features is paramount for patient counseling in clinical practice. Consequently, we aim to investigate the respective contribution of depression and anxiety level, as well as cognitive and physical trait fatigue to neuropsychological performance in MS patients and matched healthy controls.

## Materials and Methods

### Participants

Thirty patients (mean age: 46.63, range: 22–63; 15 males) with clinically definite MS (Polman et al., 2011), and 28 HC (mean age: 49.71, range: 26–63; 9 males) free from neurological or psychiatric disease, matched on age, gender and education, were included in this study after providing informed consent (***Table 1***). Patients were recruited at the specialised outpatient clinic for MS of the CHU of Liège for this study. This study was performed in accordance with the Declaration of Helsinki and was approved by the local ethic committee (approval number B707201213806). One patient was excluded from data analysis due to extremely low norm-referenced z-score obtained during neuropsychological assessment (Z-score at the executive composite score = –10.07, due to high number of errors during the Stroop task). Average education for MS patients is 12.79 years (range 6–17) and 13.64 years for HC (range 8–19). Patients had a disease duration of 13.72 years (range: 0.5–35) and an Expanded Disability Status Scale score (EDSS; Kurtzke, 1983) lower or equal to six (median: 4; range: 1–6). In the MS group, 11 people presented a RRMS, 14 a PPMS, and 4 a SPMS (see Table S1 for patient’s demographics and characteristics depending on disease course). Every patient was free from relapse for at least four weeks prior to their participation.

**Table 1:** Participant’s demographics and characteristics.


	MS PATIENTS (*N* = 29)	HEALTHY CONTROLS (*N* = 28)	*P*

**Age**, y, mean (SD)	47.48 (10.65)	49.71 (9.88)	0.42 (*t* = 0.82)

**Women**, n (%)	14 (48.3)	19 (67.90)	0.13 (*χ*^2^ = 2.24)

**Education**, y, mean (SD)	12.79 (3.33)	13.64 (2.64)	0.29 (*t* = 1.07)

**Disease duration**, y, mean (SD)	13.72 (10.27)	n.a.	n.a.

**EDSS**, median (range)	4.00 (1–6)	n.a.	n.a.


MS: Multiple Sclerosis; EDSS: Expanded Disability Status Scale.

### Cognitive testing and self-reports

Each participant underwent a standardized comprehensive neuropsychological assessment performed by a clinical neuropsychologist or a neurologist at the University Hospital of Liège (G.D., E.G. and E.L.). Cognitive examination was split into two sessions (1.5 hours approximately, with breaks provided if needed and when appropriate) to avoid triggering of fatigue and included tests of attention, verbal and visual memory, processing speed, working memory and executive functioning. Each participant filled in the French version of the Hospital Anxiety and Depression scale (HAD, Zigmond & Snaith, 1983). This self-assessed questionnaire consists of 14 items reflecting personal feelings and behaviors during the past week. Each item is quoted on an ordinal scale from 0 to 3, with seven items focusing on anxiety symptoms and the seven other on depression. A score of 11 or more in one of the two subscales is suggestive of anxiety or depressive disorder, accordingly.

Fatigue was assessed using the Modified-Fatigue Impact Scale (MFIS, Ritvo et al., 1997). This questionnaire is a shortened version of the FIS, originally developed to assess fatigue symptom in MS and validated in French (Debouverie, Pittion-Vouyovitch, Louis, & Guillemin, 2007). The 21 items (ordinal scale from 0 to 4) of the MFIS evaluate the impact of fatigue experienced in daily living during the past four weeks from which cognitive (cogMFIS), physical (physMFIS) and psychosocial fatigue scores can be derived. The psychological sub-score of the MFIS was not included in the analysis, since it is composed of only two items (score range: 0–8) and has a lower internal consistency than the two other scores (Kos et al., 2005).

### Cognitive scores

As our participant sample is heterogeneous for age and education level, raw performance was first transformed in percentiles or Z-scores on the basis of the normative sample available for each task. Next, we summarized these values in six composite scores reflecting cognitive functions that are frequently impaired in MS disease course: processing speed, working memory, verbal learning, visual learning, executive functions and attention (Bobko, Roth, & Buster, 2007). Neuropsychological tests and methods used to develop the composite scores are described below. Raw cognitive scores for both groups and corresponding norm-referenced values are presented in Appendix (Table S2).

- *Processing speed*. The raw scores for the **Digit symbol** and **Symbol search** subtests of the Wechsler Adult Intelligence Scale – III (WAIS-III) were standardized in reference to their normative sample (Wechsler, 2000) and averaged to form the processing speed composite score (corresponding to the Processing Speed Index of the WAIS-III).
- *Working memory*. The raw scores for the **Arithmetic** and the **Digit Span** subtests of the WAIS-III were standardized in reference to their normative sample (Wechsler, 2000) and averaged to form the working memory composite score (corresponding to the Working Memory Index of the WAIS-III).
- *Executive functioning*. The composite score corresponds to the average of the z-scores obtained on **phonemic and semantic verbal fluency** (Université de Liège, 2017), response time and number of non-corrected errors at **the Stroop interference condition** (Godefroy, 2007), median reaction time and total errors at the **flexibility sub-test of the Test of Attentional Performances** (TAP, version 2.3; Zimmermann & Fimm, 2010).
- *Verbal learning*. This score corresponds to the norm-referenced z-score for the sum of the five recalls at the French adaptation of the **California Verbal Learning Test** (Deweer, Poitrenaud, Kalafat & Van Der Linden, 2008).
- *Visual learning*. This score corresponds to the norm-referenced percentile (Pc) rank for the sum of the three recalls at the **10/36 sub-test of the BCcogSEP** (Dujardin, Sockeel, Cabaret, De Sèze & Vermersch, 2004).
- *Attention*. Median of response times and their standard deviation at the **auditory attention and alertness** (with signals and without signals) **sub-tests from the TAP** were transformed in percentile ranks (Zimmermann & Fimm, 2010). These percentile scores were averaged to form our composite index of attention.

### Statistics

Data used for statistical analysis are available upon request. Independent samples *t*-test assessed equality of group means for demographics, self-reports and cognitive scores. The equivalence of gender proportion between groups was tested with a Chi-squared test.

To assess multi-collinearity of variables in our statistical models, correlations between questionnaire scores (depression, anxiety, cognitive and physical fatigue) were assessed using Kendall’s tau for each group, separately.

In order to assess whether self-reported cognitive fatigue, physical fatigue, anxiety and depression are predictors of cognitive abilities in MS and in matched HC, stepwise regression analyses were performed in each group, separately, for the six cognitive scores (testing 12 models altogether, 6 per participant group). In the HC group, predictor variables consisted of cognitive and physical sub-scores at the MFIS as well as anxiety and depression scores at the HAD. The same predictor variables were considered in the patient group, along with the EDSS disability score, to control for disease progression. Since this study was designed for exploratory purpose, the statistical threshold to enter and remain in the stepwise model was set to 0.1 to be able to also observe results that show a tendency for significance, and p-values are reported without correction for multiple comparisons.

To our knowledge, no effect size has been reported in the literature regarding the link between cognition and our variables of interest. Consequently, no expected effect size could be estimated with confidence. Using G*Power 3.1.7 (Faul, Erdfelder, Buchner, & Lang, 2009) we calculated that with a power of 0.8 and an αerror of 0.05, the sample required to evidence a medium effect size (F^2^ = 0.15) of at least one predictive variable within each group would be of 55 subjects and of 25 subjects for a large effect size (F^2^ = 0.35). As 29 patients and 28 healthy subjects were included in this study, we expect to evidence large effect size. However our study might fail to evidence effects of small and medium sizes.

## Results

### Demographics

Age (*t* = 0.82, *p* = 0.42), gender proportion (*χ*^2^(1) = 2.24, *p* = 0.13) and years of education (*t* = 1.07, *p* = 0.29) did not differ between groups (***Table 1***).

### Self-reports and cognitive scores

Self-reported scores (cognitive and physical fatigue, anxiety, depression) and cognitive performance for MS patients and healthy controls are given in ***Table 2***.

**Table 2:** T-tests results for self-report measures and cognitive scores between the MS group and the healthy controls group.


	HEALTHY CONTROLS (*N* = 28)	MS PATIENTS (*N* = 29)	*T*
	
	MEAN (SD)	MEAN (SD)	

**HADS**			

Anxiety (max = 21)	6.54 (3.27)	6.66 (3.63)	–0.13

Depression (max = 21)	3.36 (2.98)	5.48 (3.96)	–2.28*

**MFIS** (max = 84)	28.00 (18.54)	42.45 (17.55)	–3.02**

Cognitive fatigue (max = 40)	13.14 (9.30)	16.10 (10.00)	–1.16

Physical fatigue (max = 36)	12.79 (8.82)	22.00 (8.36)	–4.05***

**Processing speed** (Index)	112.07 (11.10)	98.55 (18.42)	3.34**

**Working-memory** (Index)	102.04 (11.31)	99.90 (12.39)	0.68

**Executive functioning** (Z-Scores)	0.36 (0.46)	0.12 (0.75)	1.53

**Verbal learning** (Z-Scores)	0.60 (0.95)	0.26 (1.56)	0.10

**Visual learning** (Percentile)	55.39 (27.70)	46.86 (29.34)	1.13

**Attention** (Percentile)	53.46 (13.85)	39.98 (17.40)	3.23**


* *p* < 0.05; ** *p* < 0.01; *** *p* < 0.001. HADS: Hospital Anxiety and Depression Scale; MFIS: Modified-Fatigue Impact Scale; Pc: percentiles.

There was no significant difference between groups for the HAD scale anxiety score, as well as for the cognitive fatigue sub-score of the MFIS (cogMFIS). However, patients scored significantly higher on the HAD depression scale (*t* = 2.28, *p* < 0.05) and on the physical sub-scale of the MFIS (phyMFIS) (*t* = –4.05, *p* < 0.001).

Three patients (10.34%) and three HC (10.71%) reached the recommended cutoff of 11 for anxiety disorder; five patients (17.24%) and one HC (3.57%) for depression (Watson, Ford, Worthington & Lincoln, 2014). Regarding MFIS scores, normalized values (Strober et al., 2020) were above 1.5 standard deviation for 11 patients (37.93%) and 10 HC (35.71%) for the cognitive subscale, 23 patients (79.31%) and 10 HC for the physical subscale and 21 patients (72.41%) and 10 HC (37.71%) for the total score.

Regarding neuropsychological assessment, no between-group differences for executive functioning, working memory, verbal learning and visual learning scores were observed (*p* > 0.05). However, MS patients obtained lower scores for processing speed (*t* = 3.34, *p* < 0.01) and the attention (*t* = 3.23, *p* < 0.01) composite score.

Kendall’s correlations revealed significant correlations between all the self-assessment questionnaire scores in the patient group, except between anxiety and physical fatigue scores (***Table 3***). The largest association was observed between anxiety and depression (*τ*_b_ = 0.53). Disability, as measured with the EDSS, was significantly correlated to physical fatigue only (*τ*_b_ = 0.35). Regarding the control group, the largest association was observed between cognitive and physical fatigue (*τ*_b_ = 0.69), while significant correlations were also observed for depression with anxiety and with physical fatigue (***Table 4***). Despite the significance of some correlations, none of them reached a correlation of 0.8 or above. Moreover, all variable presented a tolerance above 0.1 and a variance inflation under 10, suggesting an absence of multicollinearity. Consequently, all predictor variables were kept in our statistical model.

**Table 3:** Correlation matrix (Kendall’s tau) of the predictive variables in the MS group.


	ANXIETY (HADS)	DEPRESSION (HADS)	MFISCOG	MFISPHYS	EDSS

**Anxiety (HADS)**	1.00	–	–	–	–

**Depression (HADS)**	0.53***	1.00	–	–	–

**MFIScog**	0.40**	0.35*	1.00	–	–

**MFISphy**	0.26	0.35*	0.40**	1.00	–

**EDSS**	–0.09	0.23	–0.11	0.35*	1.00


HADS: Hospital Anxiety and Depression Scale; MFIScog: Cognitive subscale of the Modified-Fatigue Impact Scale; MFISphys: Physical subscale of the Modified-Fatigue Impact Scale; EDSS: Expanded Disability Status Scale. * *p* < 0.05; ** *p* < 0.01; *** *p* < 0.001.

**Table 4:** Correlation matrix (Kendall’s tau) of the predictive variables in the HC group.


	ANXIETY (HADS)	DEPRESSION (HADS)	MFISCOG	MFISPHYS

**Anxiety (HADS)**	1.00	–	–	–

**Depression (HADS)**	0.31*	1.00	–	–

**MFIScog**	0.27	0.21	1.00	–

**MFISphy**	0.18	0.34*	0.69***	1.00


HADS: Hospital Anxiety and Depression Scale; MFIScog: Cognitive subscale of the Modified-Fatigue Impact Scale; MFISphys: Physical subscale of the Modified-Fatigue Impact Scale. * *p* < 0.05; ** *p* < 0.01; *** *p* < 0.001.

### Stepwise regression analyses

**Figure 1:**
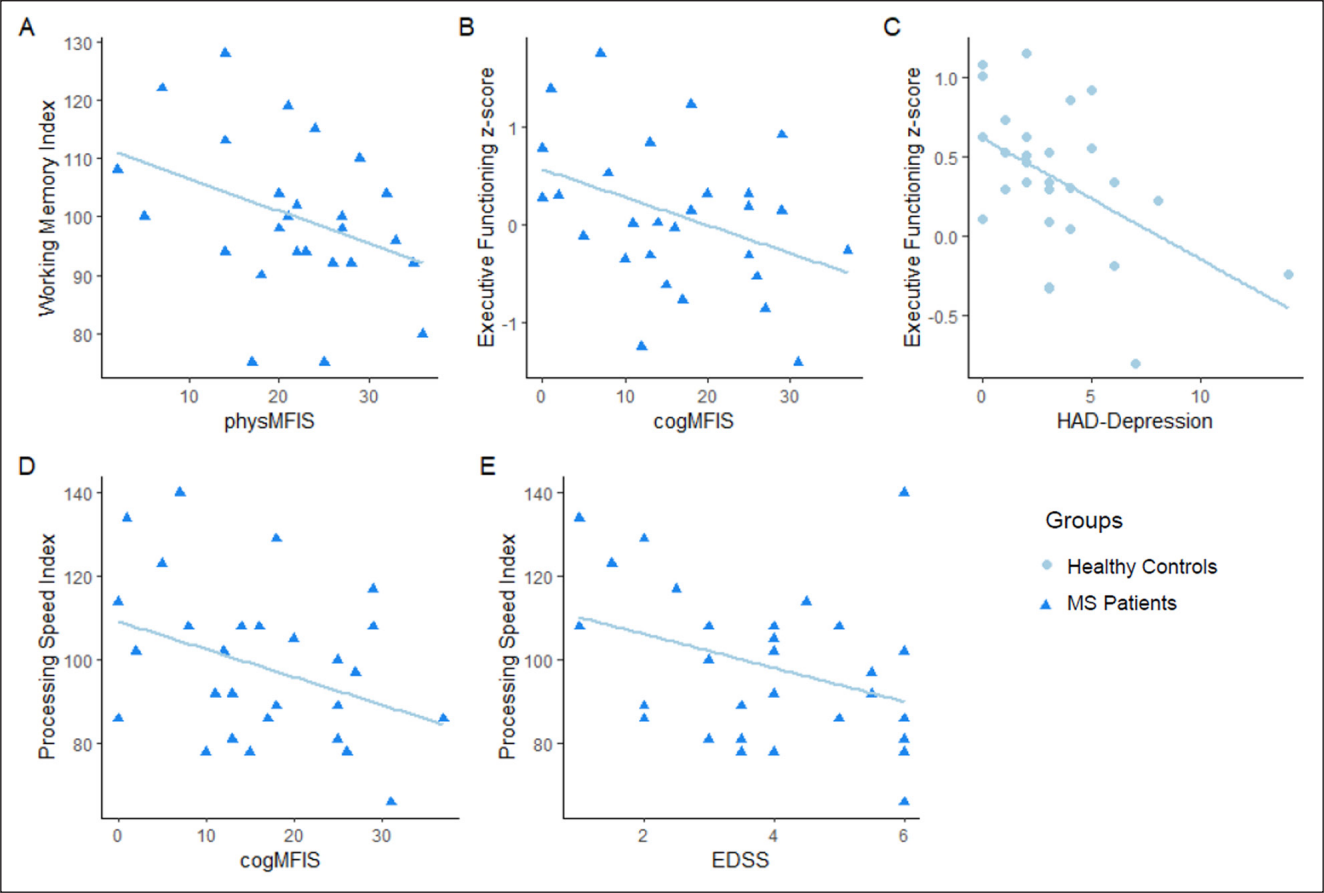
Illustration of regression lines in MS patients and healthy controls for variables included in significant models. Data points and regression lines for **(A)** working memory index depending on physical fatigue (physMFIS), **(B)** executive functioning z-score depending on cognitive fatigue (cogMFIS), **(C)** executive functioning z-score depending on depression (HAD Depression), **(D)** processing speed index depending on cogMFIS and **(E)** processing speed index depending on EDSS (Expanded Disability Status Scale) score for MS patients. Healthy controls are depicted in light blue circles, MS patients in dark blue triangles.

Statistical values for the models that reach significance are presented in ***Table 5*** and the regressions for the significant models are displayed in ***Figure 1***. In MS patients, the physMFIS score is the only significant predictor in the regression model explaining working memory performance, accounting for 14% of the variance. In the model investigating executive functioning, cogMFIS was the only significant predictor and accounted for 15% of the variance. The model predicting processing speed was significant with cogMFIS score entering as a first step variable, followed by EDSS score, altogether accounting for 28% of the variance. In the three remaining models (verbal and visual learning, attention), none of the five predictive variables were included in the model (all *p* > 0.1).

**Table 5:** Stepwise regression results (significant level for entry into the model set to 0.1).


	STEP	PREDICTOR	PARTIAL *R*^2^	MODEL *R*^2^	*F*	*P* VALUE

MS Group	Model	*F*(1, 28) = 4.41 (*p* < *0.05*) *F^2^* = 0.16				

Working Memory	1	MFISphys	0.14	0.14	4.41	0.045
Working Memory = 112.11–0.56 * MFISphys						
MS Group	Model	*F*(1, 28) = 4.88 (*p* < *0.05*) *F^2^* = 0.18				

Executive Functioning	1	MFIScog	0.15	0.15	4.62	0.041
Executive functioning = 0.57–0.03 * MFIScog						
MS Group	Model	*F*(2, 28) = 4.93 (*p* < *0.05*) *F^2^* = 0.38				

Processing Speed	1	MFIScog	0.13	0.13	5.38	0.029
	2	EDSS	0.14	0.28	5.19	0.031
Processing Speed = 127.05–0.72 * MFIScog – 4.36 * EDSS						
HC Group	Model	*F*(1, 27) = 8.40 (*p* < *0.01*) *F^2^* = 0.32				

Executive Functioning	1	HAD Depression	0.24	0.24	8.40	0.006
Executive Functioning = 0.62–0.08 *HAD Depression						


In healthy controls, executive functioning was predicted by depression score, which explained 24% of the variance.

As the composite executive score is composed of tasks recruiting different executive processes, we tentatively checked if the effects observed here are widespread across functions (initiation, inhibition and flexibility), or mainly driven by one test constituting the composite score. Results are presented in supplemental material (supplemental Tables S3 and S4). We observed that the link found between executive functioning and fatigue in MS is mainly carried-out by inhibition (Stroop task), with a lower performance for MS patients on this task. In the HC group, the link found between executive functioning and depression seems to be mainly driven by the score at the verbal fluency tests.

Additionally, as cognitive scores in the patients group was mainly explained by fatigue scores, and due to the exploratory purpose of this study, we performed supplemental analyses to investigate if the MS patients differed regarding cognition when comparing fatigued vs. non-fatigued patients (see supplemental Table S5). No between-group difference was observed.

## Discussion

The present study aimed to determine the influence of trait physical and cognitive fatigue along with mood disorders on neuropsychological performance in MS. We observed that self-reports of physical and cognitive fatigue are significant predictors of working memory and executive functions performance and processing speed, respectively. No relationship was found with anxiety and depression levels, and disability score was related to processing speed only. Cognitive performance in the healthy control group was not associated with self-reports of anxiety, depression nor fatigue, except for executive functioning and depression.

### Cognitive performance and self-report questionnaires in MS

The comparison of the composite cognitive scores showed a lower performance in the MS group, by comparison to HC, for processing speed and attention, which is consistent with the existing literature (Bobholz & Rao, 2003; Chiaravalloti & DeLuca, 2008). No difference was observed for the composite scores of working memory, executive functioning (yet supplemental analysis showed a between group difference regarding the Stroop task) and verbal and visual learning. While impairments in working memory and executive functioning are not constantly observed in MS, it is odd to observe preserved episodic memory abilities (Benedict & Bobholz, 2007; Bobholz & Rao, 2003; Chiaravalloti & DeLuca, 2008). This result can be related to the low prevalence of neuropsychological impairment in our sample, with only a third of patients showing at least one cognitive score below clinical cutoff, which is a small prevalence in view of the literature (see Chiaravalloti & DeLuca, 2008). Nevertheless, this pattern of cognitive performance is particularly interesting as it will allow to investigate the influence of fatigue and anxio-depressive symptoms on cognitive domains (or processes) that are impaired (processing speed, and attention, as well as, more tentatively, the inhibition process) or preserved (composite score of executive functions, working memory, verbal and visual learning).

Anxiety, depression and fatigue prevalence in the MS group was in line with the literature (Boeschoten et al., 2017; Weiland et al., 2015), despite the fact that we observed higher score by comparison to control for physical fatigue only. Nevertheless, it is important to note that fatigue prevalence in the control group was high, with a score above the recommended cutoff, for both physical and cognitive subscales (Strober et al., 2020) in more than one third of HC.^1^

### Relationships between cognition and fatigue

In the present study, depression and anxiety did not significantly explain cognitive performance in the patient group, contrary to fatigue level. Yet, depression significantly predicted executive abilities in healthy controls. This result is rather not surprising as the link between depression and executive abilities have widely been demonstrated (Grahek, Shenhav, Musslick, Krebs, & Koster, 2018; Nuño, Gómez-Benito, Carmona, & Pino, 2021).

It is important to note that significant correlations were obtained between anxio-depressive state and fatigue in both groups (see ***Tables 3*** and ***4***). Such associations were observed in previous studies and were attributed to overlapping symptoms between fatigue and anxio-depressive conditions, as for example, insomnia or difficulties to concentrate (Corfield, Martin & Nyholt, 2016; Greeke et al., 2017). Consequently, we consider that depression and anxiety were not main explanatory variables in our models since fatigue was a better predictor of cognition in MS. As only severe depression symptoms might impact cognition in MS patients (Golan et al., 2018), the absence of relationship could be explained by characteristics of our sample. Indeed, the mean depression score of our patients, although significantly higher than controls, remains under the validated cutoff of 11 for clinical depression (Watson, Ford, Worthington & Lincoln, 2014).

#### Physical fatigue

In the patients group, we observed an association between performance in working memory (a preserved cognitive domain in our sample) and physical fatigue only. Few studies investigated how physical fatigue relates to cognition in MS. The only one including a working memory measure found a link between physical fatigue and processing speed (SDMT and Trail Making A), but not working memory (forward and backward Digit Span) (Andreasen et al., 2019). Yet, in contrast to our study, half of the patients included in their sample had an impaired working memory score. This is particularly relevant in view of our results since neuroimaging studies of cognitively preserved MS patients showed increased activity in fronto-parietal brain networks during working memory tasks attributed to compensatory mechanisms (See Genova, Sumowski, Chiaravalloti, Voelbel & Deluca, 2009 and Rocca, De Meo & Filippi, 2016 for reviews). Additionally, it was also shown that increased brain activity observed in neurological illnesses triggers acute fatigue manifesting itself through a decrease in task performance (see Ishii, Tanaka & Watanabe, 2014 for a review), notably in the fronto-parietal network associated to working memory. Consequently, we tentatively propose that our results might reflect compensatory mechanisms, enabling MS patients to maintain a stable cognitive status despite structural brain damage, but leading to an increased subjective feeling of physical fatigue.

#### Cognitive fatigue

Moreover, our results suggest that MS patients may fail to compensate the deleterious impact of trait cognitive fatigue during the neuropsychological assessment, when tasks are resource-demanding (the inhibitory score from the executive composite) or time-constrained (processing speed composite score). The effect of cognitive fatigue on executive functioning and processing speed is well demonstrated in healthy participants (e.g. Boksem, Meijman & Lorist, 2005, 2006; Burke et al., 2018; Kato, Endo & Kizuka, 2009; Tanaka, Shigihara, Funakura, Kanai & Watanabe, 2012; van der Linden, Frese & Meijman, 2003; Wang, Trongnetrpunya, Samuel, Ding & Kluger, 2016), and also in MS patients for processing speed (Claros-salinas, Dittmer, Sehle, Spiteri & Schoenfeld, 2013; Jennekens-Schinkel, Sanders, Lanser & Van der Velde, 1988).

However, these studies used fatigue inducing protocols, and the effect of chronic trait fatigue on cognition was rarely investigated. Golan and colleagues (2018) reported an association between trait fatigue and executive functioning, but this link seemed to be mediated by depression level. With regard to processing speed in MS, some studies reported a relationship with cognitive fatigue (Andreasen et al., 2019; Diamond et al., 2008; Heesen et al., 2010) but not others (Golan et al., 2018; Morrow et al., 2009; Niino et al., 2014), with our data also suggesting an effect of trait cognitive fatigue on processing speed. In the literature, the absence of association between fatigue and processing speed is generally observed when MS participants with relatively low disability score (as assessed with the EDSS) or mainly RRMS patients are included. In our study, EDSS was indeed a predictor of processing speed, and shared a similar impact with cognitive fatigue. Taken together, disability score and cognitive fatigue were strong predictors of processing speed index.

In summary, there exists discrepancies in the literature assessing the influence of physical and cognitive fatigue on cognitive performance in MS. A possible explanation could be the influence of sample-related characteristics. For example, a differential impact of fatigue on cognition by MS subtypes has not been investigated so far. There exists evidence that cognitive deficits are more prominent (Johnen et al., 2017), and fatigue is more prevalent (Rooney et al., 2019) in the progressive forms of MS. Consequently, we could propose that fatigue gradually impacts cognition as disease progresses and brain structural alterations add up, and spread from high-level processes (e.g., executive; working memory) to more basic ones (processing speed). Further work investigating the differences between RRMS and PMS regarding fatigue modalities are greatly needed to better understand how trait fatigue impacts daily life cognition in MS.

### Mechanisms of the influence of MS-related fatigue on cognition

Here, fatigue was predictive of both impaired and preserved cognitive processes in MS, which might indicate that distinct processes are in play. We tentatively interpret fatigue associated with preserved working memory performance and executive functioning (composite score) by efficient (but costly) compensatory processes while decreased performance on processing speed and inhibition was attributed to the presence of chronic fatigue.

These two interpretations can be reconciled within the framework of the dual regulation system of central fatigue (see Ishii et al., 2014 for mental fatigue and Tanaka & Watanabe, 2012 for physical fatigue). In this model, fatigue is considered as the difficulty to initiate or sustain activity. While performing a challenging task, fatigue signal received in the central nervous system will activate an inhibition response to decrease engagement in the current activity (activation of the inhibitory system). At the same time, the deleterious effects of fatigue on activity can be compensated to maintain performance by an increased cerebral activity (activation of the facilitating system).

With respect to the present study, activation of the facilitating system to maintain cognitive performance, despite subjective feeling of fatigue, could explain the relationship observed between physical fatigue and working memory, and between cognitive fatigue and executive functioning in MS patients (Tanaka & Watanabe, 2012). Conversely, it has been proposed that an over activation of the facilitating system through prolonged and repeated mental load can lead to its dysfunction (Ishii et al., 2014). Moreover, an enhancement of the inhibitory system is prone to occur after sensitization or conditioning to central fatigue (Ishii et al., 2014). Taken together, those mechanisms are likely to induce chronic mental fatigue in patients with neurological disorders (Ishii et al., 2014), that could explain the association between trait fatigue and slowed processing speed we observe here.

When comparing MS patients according to their fatigue status, however, we did not find any significant between group differences regarding cognition (supplemental Table S5). This result suggests that cognitive status of fatigued MS patient did not considerably differ from those of non-fatigued patients in our sample. However, this absence of group differences might be attributed to the very limited sample size in this supplemental analysis as well as the cut-off used for the analysis (1.5 standard deviation from mean). Indeed, when comparing non-fatigued and severely fatigued MS patients, Pokryszko-Dragan et al. (2016) found significant differences regarding verbal and visual learning, processing speed and working memory. These differences were less clear when comparing severely fatigued patients to the moderately fatigued ones.

We do not observe an influence of trait fatigue on cognition in HC, as only depression was predictive of cognition in this group. Fatigue in healthy adults only impact on cognition when it is acute, as for example, following a fatigue induction protocol (Boksem et al., 2005, 2006; Burke et al., 2018; Kato et al., 2009; Wang et al., 2016), while, as indicated previously, in MS patients it would reflect a chronic fatigue state (Diamond et al., 2008; Ishii et al., 2014). However, due to the limited sample composing our control group, we cannot exclude the existence of subtle, moderated effects of trait fatigue on cognition in healthy adults.

## Limitations & Perspectives

Several limitations have to be pointed out in the results. First, as mentioned above, the sample used in the study is rather small considering the numerous statistical analysis performed and we did not correct for multiple comparisons. As previously indicated, the present study was designed in an exploratory perspective. Thus, our results should be taken with caution, as a first step toward the understanding of the differential effects of cognitive and physical fatigue on cognition. In this sense, replication by other studies is greatly needed, especially to investigate potential effects of smaller size with a larger sample and statistical power. Nevertheless, our results do support the existence of distinct influences of cognitive and physical fatigue on cognition in MS.

Second, in the present study, we do not directly compare the two groups regarding the multiple regressions analysis. Therefore, we only discussed the results found in each group separately and do not state that the results observed are specific to MS. As already mentioned, the lack of association observed between fatigue and cognition in the healthy controls group could also be attributed to the sample size.

Third, as discussed in the introduction section, many other variables are susceptible to influence fatigue and cognition in MS. This is the case, for instance, of sleep disorders. Sleep disorder are frequently reported in MS (Sakkas, Giannaki, Karatzaferi, & Manconi, 2019; Veauthier, 2015), and it has been often associated to fatigue symptom (Attarian, Brown, Duntley, Carter, & Cross, 2004; Galland-Decker, Marques-Vidal, & Vollenweider, 2019; Paucke, Kern, & Ziemssen, 2018). Unfortunately, we do not have access to sleep quality score in our sample. However, it would be of great interest to see if sleep disorders can mediate the link we observe between fatigue and cognition as it is a common cause of secondary fatigue in MS. Medication is also a variable that is known to influence fatigue, mood, and/or cognition. Controlling for medication is highly challenging and rarely done in the MS literature. Although we did not control for medication in this study, we graphically represented participants in the MS group depending on their medication status for our main results (see supplemental Figure S1). A visual inspection of the graphs does not show any cluster related to medication status.

Differentiating the effects of primary fatigue (specific to MS and due to cerebral alterations) from secondary fatigue (triggered by other symptoms that are frequent in MS, but not specific to the disease) might provide valuable information regarding the specificity of MS-related fatigue. Factors leading to secondary fatigue have been largely studied and identified (see Krupp, 2010 and Penner & Paul, 2017 for a review). However, distinguishing their effects from those of primary fatigue is challenging. Yet this seems particularly relevant from a clinical viewpoint. As secondary fatigue can be specifically tackled (for instance by improving sleep quality), it is crucial to determine if this symptom has an impact on cognition.

Finally, in this article, we investigated which variables could predict cognition in MS. The reversed question could also be of great interest: Does cognitive impairment predict fatigue? Several studies tried to answer this question, mainly by means of fatigue induction protocols, but the directionality of the link between cognition and fatigue is still unclear. As developed earlier, both mechanisms could be at play, with fatigue worsening cognition, and effort to compensate cognitive impairment triggering fatigue. Longitudinal and functional MRI protocols are very promising to elucidate this question.

## Conclusion

Fatigue predict cognition in MS beyond anxio-depressive symptoms, with a distinct influence of trait fatigue on cognition depending on its modality. Our results foster the need to consider fatigue as multidimensional in further research works. Indeed, it seems that depending on its modality, fatigue impacts distinct cognitive processes in MS.

## Additional File

The additional file for this article can be found as follows:

10.5334/pb.1125.s1 Supplementary Material. Tables S1–S5 and Figure S1.

## Footnotes

While we expected higher fatigue level in our patients by comparison to controls, we consider that our regression analyses allows to discuss the effect of pathological and normal fatigue on cognition, as MS patients describe fatigue related disease as different from what experienced prior to the disease (Krupp et al., 1988; Murray, 1985).
