# Supplementary material for: The Complex Interplay Between Trait Fatigue and Cognition in Multiple Sclerosis
Source: Psychol Belg. 2022 Mar 16;62(1):108–22. doi: 10.5334/pb.1125 (PMC8932362; doi:10.5334/pb.1125)
Supplement: Supplementary Material. — Tables S1–S5 and Figure S1. [file pb-62-1-1125-s1.pdf]

Supplemental Material

Table S1: MS patient's demographics and characteristics depending on disease course

	RRMS (*n* = 11)	PPMS (*n* = 14)	SPMS (*n* = 4)	All MS patients (*n* = 29)
**Age**, y, mean (SD)	39.27 (8.36)	52.29 (9.52)	53.25 (5.74)	47.48 (10.65)
**Women**, *n* (%)	6 (54.55)	4 (28.57)	4 (100)	14 (48.3)
**Education**, y, mean (SD)	14.36 (2.73)	11.14 (3.44)	14.25 (1.71)	12.79 (3.33)
**Disease duration**, y, mean (SD)	10.45 (10.57)	13.79 (8.95)	22.50 (11.12)	13.72 (10.27)
**EDSS**, median (range)	2.00 (1 - 5.5)	4.75 (3 – 6)	4.50 (3.5 – 6)	4.00 (1 - 6)

MS: Multiple Sclerosis; EDSS: Expanded Disability Status Scale; RRMS: Remitting Relapsing Multiple Sclerosis; PPMS: Primary Progressive Multiple Sclerosis; SPMS: Secondary Progressive Multiple Sclerosis

Table S2: Raw cognitive scores for both groups and corresponding norm-referenced values

	Healthy controls (*n* = 28)		MS Patients (*n* = 29)	
	Raw score mean (SD)	Norm-referenced value mean (SD)	Raw score mean (SD)	Norm-referenced value mean (SD)
**Processing Speed**		*Standard Score*		*Standard Score*
Digit symbol Coding	78.32 (12.76)	12.46 (2.08)	60.48 (19.83)	8.93 (3.56)
Symbol Search	35.50 (7.45)	11.86 (2.29)	31.52 (9.69)	10.59 (3.24)
**Working Memory**		*Standard Score*		*Standard Score*
Arithmetic	15.11 (4.30)	10.93 (2.87)	15.10 (2.99)	10.59 (2.04)
Digit Span	15.82 (3.65)	9.61 (2.57)	15.59 (3.78)	9.59 (2.77)
**Executive functioning**		*z-score*		*z-score*
Phonemic verbal fluency (2 min.)	23.93 (7.32)	0.25 (0.98)	23.17 (6.35)	0.28 (1.21)
Semantic verbal fluency (2 min.)	34.79 (9.67)	0.46 (1.21)	31.45 (7.51)	0.37 (1.46)
Interfering Stroop: RTs time (s)	103.20 (21.91)	0.27 (0.88)	122.70 (24.07)	- 0.51 (1.13)
Interfering Stroop: Errors	0.50 (1.29)	0.10 (1.01)	0.34 (0.77)	0.12 (0.83)
Flexibility reaction time: Median time (ms)	668.1 (132.50)	0.86 (0.87)	894.20 (435.10)	0.01 (1.15)
Flexibility errors	3.36 (4.57)	0.23 (0.85)	3.59 (6.14)	0.37 (1.01)
**Verbal learning**		*z-score*		*z-score*
CVLT: sum of 5 recalls	61.79 (9.92)	0.60 (0.95)	58.28 (12.19)	0.26 (1.56)
**Visual learning**		*Percentile*		*Percentile*
10/36: sum of 3 recalls	19.36 (5.24)	55.39 (27.70)	17.76 (5.38)	46.86 (29.34)
**Attention**		*Percentile*		*Percentile*
Auditory attention reaction time (TAP): Median time (ms)	506.50 (84.84)	47.68 (29.20)	550.30 (84.96)	33.48 (26.06)
Auditory attention reaction time (TAP): Standard deviation	79.25 (26.01)	52.00 (23.34)	94.38 (40.55)	41.28 (31.26)
Alertness reaction time without signal (TAP): Median time (ms)	242.10 (25.64)	38.54 (20.77)	283.10 (123.6)	28.03 (21.60)
Alertness reaction time without signal (TAP): Standard deviation	36.64 (19.67)	69.71 (26.94)	43.21 (28.52)	51.76 (28.92)
Alertness reaction time with signal (TAP): Median time (ms)	242.40 (28.45)	48.64 (22.71)	276.20 (104.3)	30.28 (21.12)
Alertness reaction time with signal (TAP): Standard deviation	33.29 (18.50)	64.18 (30.47)	43.45 (24.03)	55.03 (27.09)

Table S3. T-tests results for the differences in specific executive scores in the MS and the healthy controls groups

	Healthy controls (n = 28)	MS Patients (n = 29)	
	Mean (SD)	Mean (SD)	t
**Fluency**	0.36 (0.97)	0.33 (1.19)	0.10
**Stroop**	0.18 (0.70)	-0.19 (0.66)	2.08*
**Flexibility**	0.54 (0.56)	0.19 (0.95)	1.71

*$p < 0.05$; MS: Multiple Sclerosis; Fluency: mean of z-scores for phonemic and semantic fluency; Stroop: mean of z-scores for reaction time and errors; Flexibility: mean of z-scores for median reaction time and errors.

Table S4: Significant results of analysis on the three components of the executive score (Verbal fluency, Stroop and Flexibility).

	**Step**	**Predictor**	**Partial R^2**	**Model R^2**	***F***	***p* value**
MS Group	Model	$F(1, 28) = 8.47$ ($p < 0.01$) $F^2 = 0.36$				
Stroop	1	MFIScog	0.24	0.24	8.47	0.007

*Stroop = 0.33 – 0.03 * MFIScog*

HC Group	Model	$F(1, 27) = 7.38$ ($p < 0.05$) $F^2 = 0.28$				
Fluency	1	HAD Depression	0.22	0.22	7.38	0.012

*Fluency = 0.87 – 0.15 * HAD Depression*

HC Group	Model	$F(1, 27) = 3.22$ ($p = 0.08$) $F^2 = 0.12$				
Stroop	1	HAD Anxiety	0.11	0.11	3.22	0.084

*Stroop= 0.65 – 0.07 * HAD Anxiety*

In MS, we observe only a link between inhibition (Stroop task) and cognitive fatigue. In healthy controls, verbal fluency is associated to depression, and we observe a tendency for an association between performance at the Stroop task and anxiety.

Table S5: Between groups differences in composite cognitive scores in the MS group (fatigued vs. not fatigued).

	**Cognitively fatigued MS patients (*n* = 13)** Mean (SD)	**Not Cognitively Fatigued MS patients (*n* = 16)** Mean (SD)	***p* (T-test)**
Working Memory	99.62 (8.83)	100.13 (14.98)	0.915
Processing Speed	96.39 (17.22)	100.31 (19.71)	0.577
Visual Learning	50.62 (29.56)	43.81 (29.75)	0.544
Verbal Learning	-0.04 (1.91)	0.50 (1.20)	0.709[a]
Attention	41.60 (17.30)	38.66 (17.93)	0.659
Executive Functioning	-0.01 (0.69)	0.21 (0.80)	0.443

	**Physically fatigued MS patients(*n* = 23)** Mean (SD)	**Not Physically Fatigued MS patients (*n* = 6)** Mean (SD)	***p* (T-test)**
Working Memory	97.87 (9.70)	107.67 (18.83)	0.084
Processing Speed	96.13 (17.80)	107.83 (19.35)	0.170
Visual Learning	46.44 (30.33)	48.50 (27.73)	0.978[a]
Verbal Learning	0.12 (1.69)	0.77 (0.75)	0.747[a]
Attention	39.34 (18.25)	42.42 (14.86)	0.707
Executive Functioning	0.13 (0.72)	0.04 (0.92)	0.801

The patients group was split in two according to the normative value of the MFIS (fatigued: z-score > 1.5, not fatigued: z-score < 1.5). This was done for the cognitive sub-scale of the MFIS and the physical sub-scale. No between group difference was observed regarding cognitive scores.

[a] Mann-Whitney test due to deviation from normality

Figure S1: Representation of the association between cognition and fatigue in the patient group according to medication.

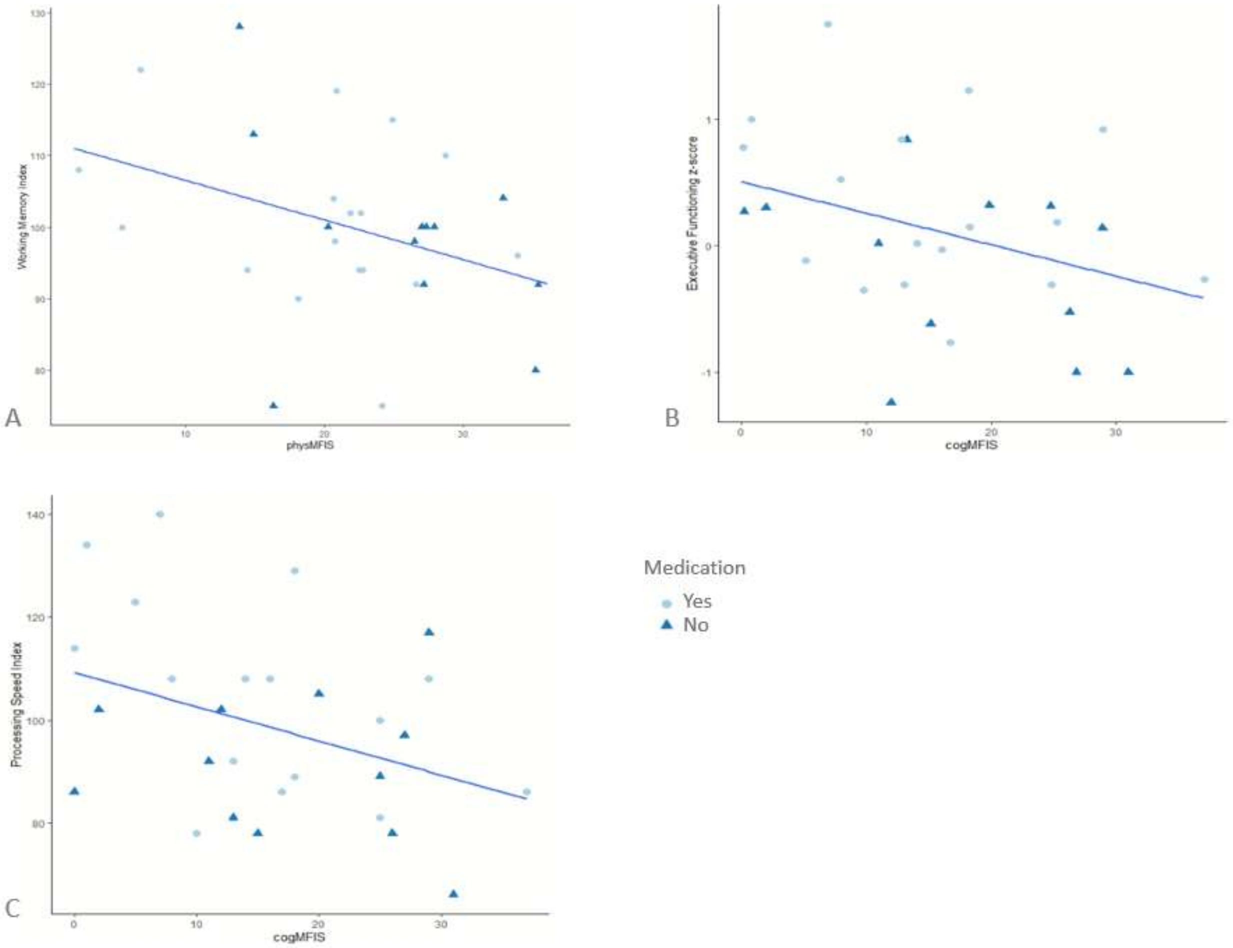

Graphical assessment of the effect of medication on our main results (A: working memory index and physical fatigue (physMFIS); B: executive functioning z-score and cognitive fatigue (cogMFIS); C: processing speed index and cogMFIS). Patients with a treatment potentially influencing fatigue, mood and/or cognition are depicted with dark blue
